# The impact of the COVID-19 pandemic on people who inject drugs accessing harm reduction services in a rural American state

**DOI:** 10.1186/s12954-022-00660-2

**Published:** 2022-07-22

**Authors:** Kinna Thakarar, Michael Kohut, Rebecca Hutchinson, Rebecca Bell, Hannah E. Loeb, Debra Burris, Kathleen M. Fairfield

**Affiliations:** 1 Center for Interdisciplinary Population and Health Research/Maine Health Institute for Research, 509 Forest Ave, Portland, ME USA; 2 Department of Medicine, Maine Medical Center, 22 Bramhall Street, Portland, ME USA; 3 Tufts University School of Medicine, 145 Harrison Ave, Boston, MA USA

**Keywords:** Injection drug use, Marginalized and mobile populations, Social and economic issues

## Abstract

**Background:**

The impact of public health policies during the COVID-19 pandemic on people who inject drugs (PWID) has varied across regions. In other countries, recent research has shown that PWID access to harm reduction services, despite rapid adaptations, has been negatively impacted. Our study describes these impacts in a rural state.

**Methods:**

We conducted semi-structured interviews with PWID, community partners, and healthcare providers in the rural state of Maine (USA). We explored how changes made during the pandemic impacted access to harm reduction services, including basic services (i.e., shelter), syringe service programs, safe drug supply, low barrier treatment, and peer support. Interviews were analyzed using the framework method to apply Penchansky’s model of access, with Saurman’s modification, which includes six dimensions of access—accessibility, availability, acceptability, affordability, accommodation, awareness.

**Results:**

We interviewed thirty-six stakeholders (*N* = 9 community partners, *N* = 9 healthcare providers, *N* = 18 PWID). Policies such as mobile outreach expansion, mail delivery of equipment, and relaxed telemedicine regulations facilitated accessibility to syringe service programs and low barrier buprenorphine treatment. Public health policies, such as social distancing and screening policies, reduced contact, which subsequently reduced acceptability and awareness of many services. Elimination of the one-for-one needle exchange in some areas increased, acceptability (i.e., perception of service), and affordability for PWID. However, some areas actually began enforcing a one-for-one needle exchange policy, which reduced affordability, acceptability, and awareness of services.

**Conclusions:**

Changes resulting from the COVID-19 pandemic have impacted all dimensions of access to harm reduction services among PWID. While some barriers to harm reduction services were unavoidable during the pandemic, we found that specific policy decisions mitigated service barriers, while other policies exacerbated them. Relaxing needle exchange policies were particularly helpful in facilitating access to harm reduction services by giving community organizations flexibility to adapt to the evolving needs of PWID. These results can inform policies and service delivery to optimally mitigate the negative impacts on PWID during, and beyond, the pandemic.

**Supplementary Information:**

The online version contains supplementary material available at 10.1186/s12954-022-00660-2.

## Introduction

The impact of the COVID-19 pandemic on how people who inject drugs (PWID) access harm reduction services, particularly in rural areas of the USA, is poorly understood. Harm reduction services, such as syringe service programs (SSPs), basic services (i.e., shelter, food, restrooms), safe supply, peer support, and low barrier buprenorphine treatment play important roles in mitigating adverse impacts of drug use, such as infections and overdoses [1–5]. Prior to COVID-19, studies identified barriers to harm reduction services, including logistics challenges (i.e., transportation and scheduling), fees, fear of law enforcement, concerns about confidentiality, and lack of awareness [6–9]. However, disruptions in services during the pandemic are likely to have created new barriers, and exacerbated existing challenges, to service access [10, 11]. In other countries, recent research has shown that PWID access to preventive services, despite rapid adaptations, has been negatively impacted [12, 13].

Evidence is emerging on the impacts of the COVID-19 pandemic on PWID in different regions of the USA. Interviews with people who use drugs (primarily methamphetamine), in rural communities in Oregon revealed limited access to SSPs during the pandemic [14]. Another recent case report described the successful implementation of low-barrier buprenorphine treatment through telemedicine and street outreach in an urban setting in Boston [15]. In New York City, SSP utilization and naloxone possession sharply declined in one study, while a flexible methadone-dosing may have helped retain individuals in care [16]. Our study adds to the existing literature by paying particular attention to multiple dimensions of access, as well as how specific policies have impacted those dimensions. In addition, we present novel results from northern New England; prior studies may not be generalizable to a state like Maine, which is predominantly a rural state where distance to services has been identified as a barrier to accessing harm reduction services [17]. It is also an area where fentanyl is the driving cause of the overdose crisis, thus the frequency of injection and need for harm reduction services may differ from other parts of the country [18]. Early in the pandemic, Maine also had a lower number of cases of COVID-19 compared to more urban areas [19], in part likely due to population density, as well as robust public health response [20]. Like other parts of the USA and other countries, Maine has seen an increase in individuals seeking treatment for substance use disorders. While there has been a 43% increase in buprenorphine prescriptions over the past three years and expansion of opioid use disorder treatment to justice-involved populations, there is still an unmet need in both preventive and treatment services in Maine [21, 22].

**Fig. 1:**
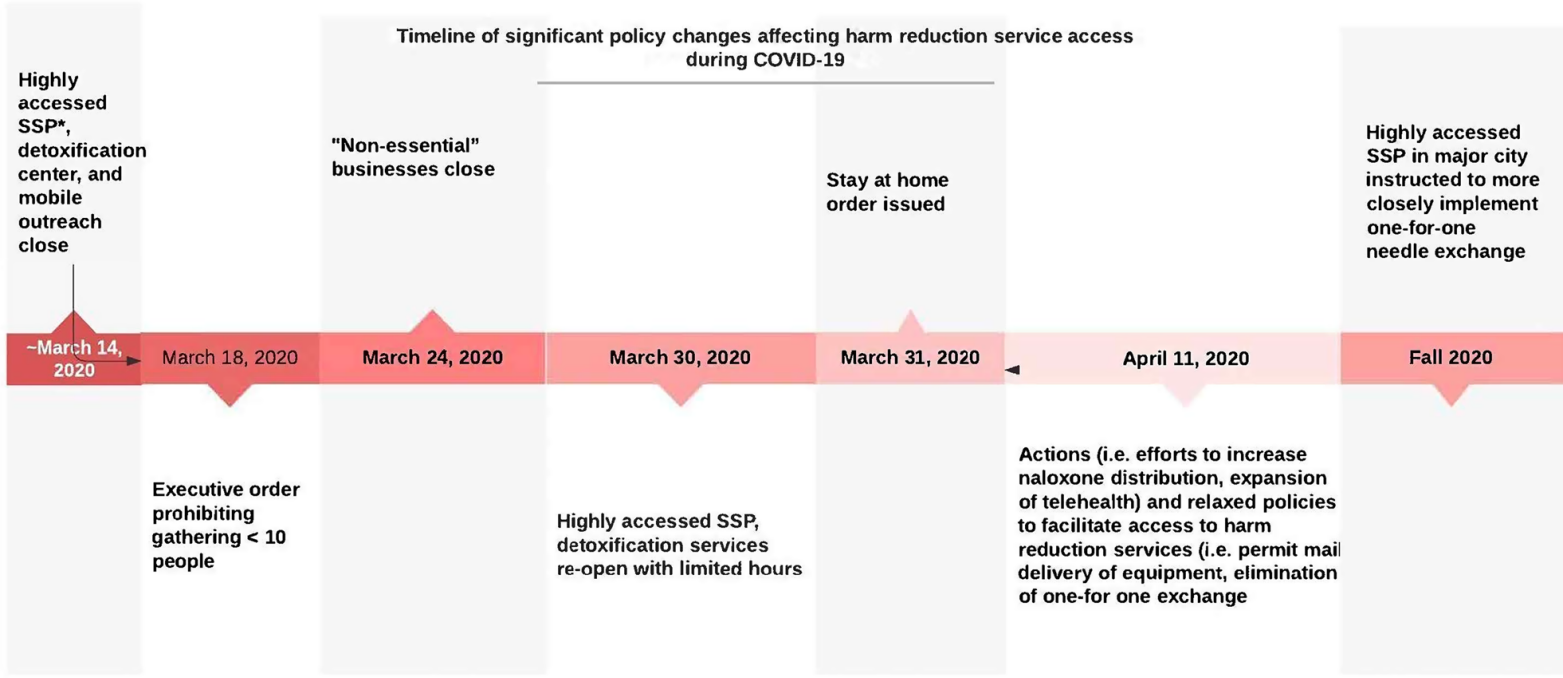
Timeline of policy changes

In Maine, an emergency shut-down order was declared on March 31, 2020, shortly after the first presumptive COVID-19 case was identified [23]. At that time, there were eleven SSP sites in Maine serving 5730 SSP enrollees [24]. Some harm reduction services, including SSPs, were temporarily closed and then reopened with limited hours and restricted access to indoor sites [25]. In order to minimize contact, medical visits were conducted through telemedicine when possible, particularly in rural areas of the state [26]. An executive order was later issued to relax some policies, such as a temporary elimination of one-for-one needle exchange in favor of free provision of needles, and permission to mail safe injection supplies directly to clients. However, some of these relaxed policies, specifically elimination of the one-for-one needle exchange expectation, were not widely implemented in high population density areas such as Portland, Maine, partly due to anecdotal concern for syringe litter [27, 28] (Fig. 1). A summary of these harm reduction service changes is presented in Table 1.

**Table 1:** Summary of harm reduction service changes during COVID-19

Type of harm reduction service	Summary of service changes during COVID-19
Syringe service programs (SSP)/safer use equipment	One highly accessed SSP, closed temporarily early in the pandemic, then re-opened with limited hours Several on-site SSPs reported equipment shortage early in the pandemic One on-site SSP shifted to outdoor location Temporary allowance of the mail and mobile delivery of equipment Temporary elimination of the one-for-one needle exchange policy
Safe drug supply	Reports of more contaminated supply People who inject drugs report purchasing drugs from new dealers Increased drug use in setting of contaminated supply
Peer support	Closure of in-person services from a highly accessed recovery center in the state COVID-19 protocol restrictions result in outreach workers being unable to connect in person with justice-involved individuals
Basic services (shelter, food security, restrooms	COVID-19 screening (e.g., temperature checks), testing, physical distancing policies and quarantine protocols in shelters Closure of several on-site food services; shift to mobile services Lack of access to public restrooms
Low barrier medication for opioid use disorder treatment	Clinic and protocol changes as a result of relaxed telemedicine regulations

In this study, we examine how changes related to COVID-19 impacted access to important harm reduction services for Maine residents. We used the model of access proposed by Penchansky and Thomas, [29], later modified by Saurman [30] as a theoretical framework to identify issues of access along six dimensions: *awareness, accessibility, availability, acceptability, affordability, and accommodation*. The model, which has been used in prior health service-related studies [31–33], recognizes that people cannot access a service unless six basic considerations are met: First, that people who use services are *aware* the service exists and know how to access it. Second, that people are able to reach the service in some way (*accessibility*). Third, that the site of service has all necessary supplies *available*. Fourth, that people who desire the services perceive the site and provision of services to be *acceptable*. Fifth, that people have the necessary resources to *afford* the service. Sixth, services are provided in a way that *accommodates* client needs. A disruption in *any* dimension of access to a harm reduction service means people who inject drugs will have reduced access to the service. Our study describes how various changes during the COVID-19 pandemic impacted each of these dimensions, including facilitators and barriers to harm reduction service access.

## Methods

### Recruitment

This study was approved by the MaineHealth Institutional IRB board. Informed consent was obtained verbally for all study participants prior to the interview. We conducted semi-structured interviews with a convenience sample of people who have injected drugs in the past year, and purposive samples of community partners and healthcare providers in Maine. We identified service providers based on their connection to major forms of harm reduction and substance use disorder treatment in the state through professional networks. We broadly included providers of essential services (such as shelter and food services), syringe service programs, low barrier medication for opioid use disorder, detoxification, and peer support. PWID participants were recruited by flyers, which community partners either shared with their clients throughout the state and/or were posted in areas frequented by people experiencing homelessness in Portland, Maine. Community partners included SSP staff, urban shelter and detoxification staff, and peer support outreach workers. Many of the community partners, such as SSP staff, serve rural populations. Healthcare providers included case managers, nurse practitioners, physicians, social workers, and pharmacists who work directly with people who use drugs. Providers were recruited from a variety of settings, including a low barrier urgent care clinic, rural emergency department, and outpatient settings.

### Study design

The inclusion of both service users and service providers (community partners and healthcare providers) ensured the inclusion of diverse perspectives and reduced blind spots associated with one group. We sought representative service providers from all SSPs in the state and from buprenorphine treatment providers serving areas throughout Maine. We discontinued recruitment of PWID after nine months to ensure participants were providing perspectives on a relatively discrete set of circumstances, which were rapidly changing.

Semi-structured interview guides (Additional file 1: Appendix) were developed for each participant category to reflect their specific knowledge. Interview guides for service providers focused on how changes during the pandemic affected service delivery access, as well as the health of clients and patients. Interview guides for PWID focused on personal experiences with drug acquisition, harm reduction and treatment services during the pandemic. To avoid bias through the acquiescence effect [34], rather than explicitly asking about each access dimension in the interview guide, we presented access as broadly as possible. As such, participants could provide information that impacted how people could or could not gain access to service.

PWID and community partners were offered a $25 gift card in appreciation of their willingness to speak with the study team and time spent in the interview. Since phone access was required for participation, if needed, we provided PWID a $10 phone card, and community partners were willing to provide phones for participants when feasible.

### Data collection

Between June 2020 and February 2021, one researcher (MK) conducted the interviews by phone. Interviews with PWID participants lasted up to 30 min, and interviews with service providers lasted 30–60 min. Interviews were digitally recorded and professionally transcribed. One analyst (MK) read transcripts and deleted any information that could be used to identify participants.

### Data analysis

Our analytical goal was to identify and describe issues of access in harm reduction across the state. We thus aimed for the greatest breadth of perspectives and adopted a “hermeneutics of faith”; this approach takes individuals at their word and “gives voice” to their concerns, without efforts to problematize their narratives or decode meaning “behind” the text [35]. Thus no triangulation was used to verify the accuracy of a claim. Issues of access were identified whether they were mentioned by a service provider or a person using drugs. We used MAXQDA, a qualitative analysis software package, to assist with analysis. Four analysts with distinct backgrounds—MK, RB, HL, KT—separately read and open-coded portions of the transcripts, focusing attention on how access to services were impacted, then met to discuss. MK and KT developed a coding system, which they systematically applied to all transcripts. To clarify the concept of access, we adopted Saurman’s version of Penchansky and Thomas’ model of access [29, 30]. We developed categorical codes to capture each dimension of access, and used the framework method [36] to organize and rapidly analyze the information provided by service providers and service users. Our framework was organized with rows for specific harm reduction services and columns for facilitators and barriers to access, split into the six dimensions of access.

To avoid missing relevant content, two analysts (MK, KT) independently applied the categorical codes to all the transcripts and compiled results in tables, based on the framework method. Resulting tables were combined and disagreements in coding were discussed and reconciled by consensus. Data regarding dimensions of access, facilitators and barriers, and representative quotes were then presented in table format to the research team for further discussion and revision.

## Results

The sample included 18 PWID and 18 service providers (9 community partners; “CP”, and 9 healthcare providers; “Pr”; total *N* = 36). Most of the PWID participants (*n* = 15) were actively using drugs, whether or not they were engaged in treatment with medication for opioid use disorder (MOUD), and many (*N* = 11) were unhoused at the time of the study interview (Additional file 2: Table S1). We also interviewed 6 community partners representing four of the largest SSPs in Maine. Provider participants included physicians, nurse practitioners, and pharmacists.

Through interviews we identified numerous changes to how harm reduction, treatment and basic medical services were provided during the first year of the COVID-19 pandemic in Maine. These changes are summarized in Table 1. Below, we present how various changes impacted each of the six dimensions of access to harm reduction services (Table 2).

**Table 2:** Facilitators and barriers to harm reduction services, stratified by dimensions of access

Dimension of access	Description of dimension	Facilitators	Barriers
Accessibility^b^	Getting to service (i.e., location, transportation issues)	Mobile SSPs^a^ Outdoor services Mailing equipment	COVID-19 screening No cell phone coverage Inclement weather Masking requirements on buses Changing locations
Availability^b^	Supply and demand	Relaxed policies SSPs sharing supplies	Stricter adherence to 1:1 needle exchange Increased drug use Lack of safe supply
Acceptability^b^	How clients perceive service	Face to face outreach Trust established before pandemic Relaxed policies Mobile outreach	Masking Social distancing policies (i.e., waiting in line) Stigma Lack of trust/difficult to establish trust under lockdown Law enforcement
Affordability^b^	Ability of clients to pay for service	Relaxed policies (i.e., elimination of 1:1 needle exchange)	Unemployment Higher drug costs Requiring one-for-one needle exchange
Accommodation^b^	Hours of service provision, structure of services, facilities	Community resilience/Staff working more hours COVID-19 screening	Limited service hours Appointment-only for health services
Awareness^c^	Communication and information about services	Outreach Flyers mailed with supplies	Miscommunication around changing policies Changing locations Restrictions on outreach workers in certain locations (i.e., jails)

^a^SSP, syringe service program

^b^Five dimensions of access described by Penchansky and Thomas

^c^Sixth dimension of access described by Saurman

### Barriers that reduced awareness of harm reduction services

The most fundamental dimension to accessing harm reduction services is awareness about how it can be accessed. With closures, shifting locations, new policies, and other changes, many service-users lacked information needed to access services. As noted, early in the pandemic, a highly accessed SSP in Portland was forced to shut down because of a COVID-19 case and then hours were restricted. Community partners noted that many clients of the SSP were not informed about the nature of the closure, and were unaware it had reopened:

“There's also a perception that places are closed, even if they're open. So, we try to provide information to our patients that, okay, the needle exchange was closed for a period of time, but they are open again, and here are the times when they are open” (Pr 1)

One PWID expressed frustration around unpredictable locations of basic services:

“Right now my feet have blood blisters all over them from just constant walking. Just making it to a place where they serve food can be hard and all of those soup kitchens are closed. So you have to nail down when the good shepherd food trucks are going to come by…it’s definitely affected our ability to eat.” (PWID 5)

Changes around the one-for-one needle exchange policy were also confusing for many PWID and community partners. For example, the executive order temporarily eliminated the one-for-one needle exchange policy in the state, but a few months into the pandemic, the city of Portland was instructed to enforce the one-for-one needle exchange policy. Several PWID who accessed the Portland SSP found the policy change both confusing and detrimental to their health. The reported confusion demonstrates that efforts to communicate these various changes were unsuccessful. One community partner suggested these issues predated the pandemic:

“My biggest grievance, I think, with the state […] is that it doesn't do a good enough job of communicating with drug users.” (CP 2)

Lacking a robust system of communication to reach people who inject, organizations relied on informal channels and direct relationships between service providers and service users to relay information. Unfortunately, COVID-19 policies that limited visitors to correctional facilities and other spaces also impacted the ability of some community partners do in-person outreach in those locations. As a result, they reported missed opportunities to connect with PWID and link them to harm reduction services.

“Previously, I was just able to go to a jail and work on those values, […] So a lot of that work was being established there. But now, because I'm not able to go in there and as people are coming out, I actually don't know what are the different services that are lacking.” (CP 05)

### Facilitators that increased awareness of harm reduction services

Many community partners increased their outreach efforts to be able to connect with more dispersed populations:

I think we have a lot of recognition that there are people for whom this isn't a great form of connecting which is why we've been really maintaining our in-person outreach in the places that we have those programs to connect with primarily people who are unhoused, who are camping in the woods, who are really living in a way that doesn't facilitate doing a FaceTime with us. (CP 3)

Community partners attempted to share information when possible, informing clients about other services that were available during brief contact or including contact cards in bags distributed with safe injection supplied. Additionally, because of the temporary executive order that allowed for mail delivery of drug equipment, several mobile SSPs were able to mail flyers advertising their services. SSP clients that requested naloxone for overdose prevention received a flyer in the mail alerting them that they could contact the SSP for other equipment as well. This approach was found to increase awareness and reduce stigma around accessing safer use equipment. As more mobile SSPs set up in outdoor spaces, their presence also increased awareness about accessing safer use equipment.

Other facilitators that increased awareness to harm reduction services, specifically low barrier buprenorphine treatment, involved services simply staying open and visible outdoors. Two providers from a low-barrier urban clinic started delivering services outdoors early in the pandemic, which demonstrated to community members their availability:

“We made a decision as a program early on that we were going to remain open as long as we could. And be available to support patients in the community...with additional safety measures in place...” (Pr 1).

### Barriers that reduced acceptability of harm reduction services

Some COVID-19 policies made particular harm reduction services less acceptable to PWID. Many service providers emphasized the ways that regular, face-to-face interactions allowed them to establish trusting relationships with their clients. New restrictions often prevented them from being present in the same space:

“…in terms of building that relationship for me has been consistency and has been presence. […] And so I think not having that face-to-face, to me has meant a lack of consistency and a lack of physical presence to build that baseline trust with each other, which then causes a lot of gaps in the services that people need.” (CP 5).

These relationships between clients and providers are also relevant to understanding difficulties adjusting to changing sites among PWID. Asked whether there are alternative services PWID can use, one community partner explained:

“Yes, they do have them, but once again, sometimes with [PWID], it's all about trust and having that rapport with folks. So, some folks are very hesitant to engage with new providers, even though we try to encourage that and help to make referrals and all those sorts of things. It still can be a barrier.” (CP 6)

While COVID-19 screening (e.g., temperature checks), testing, physical distancing policies and quarantine protocols were acceptable to some people, COVID-19 protocols also reduced acceptability to many participants seeking harm reduction services. For several reasons, including fears of COVID-19 testing and possible quarantine, PWID participants also preferred to avoid the shelters. Many stayed in tents, while others stayed in hotels or cars.

Service providers at mobile SSPs reported that guidelines around physical distancing, specifically staying six feet apart and only allowing gatherings of ten or fewer people, reduced acceptability of services:

“They get really frustrated…you have to tell them ‘Hey, there’s 10 people here…you got to wait down the street…a lot of people have gotten so frustrated that they just left and they were like ‘ I don’t got time for this.’ And then, where are they going without their safe using equipment?” (CP 1)

Enforcement of the one-for-one syringe policy in Portland impacted access in a variety of ways detailed in each section. One impact was reduced acceptability, as established relationships between a Portland SSP and its clients were undermined:

“It wasn’t like this before…It was just like ‘I want you to be safe. Take as many as you need to be safe.’…I feel like, ‘Why are they changing this all of a sudden? Do you want people safe or do you want health [problems]…There’s going to be a rise in infections and HIV, hepatitis C. Why are we regressing in this now?” (PWID 17)

Interactions with law enforcement in the setting of the one-for-one needle exchange policy in Portland specifically reduced access to safer use equipment. At the time of the study, due to regulatory barriers such as the requirement to carry less than 11 hypodermic needles, participants carrying more than the limit of safer use equipment reported issues with law enforcement:

“…people are being given a hard time by the police a lot more…they don't necessarily get arrested if they have more than 10 needles, but they get a ticket or they'll get a summons and they'll have to go to court. Or the police will just confiscate their stuff sometimes…It ties in with this whole criminalization of being homeless, as well as criminalization of using drugs. “ (CP 7).

Though not reported as a barrier exacerbated by the pandemic specifically, several PWID participants also felt that stigma was a barrier to accessing safer use equipment outside of SSPs. For example, PWID in rural areas experienced stigma when buying unused injection equipment and naloxone at community pharmacies:

“…every time I go, the pharmacies are trying to say they don't have needles, and blah, blah, blah, and it's just like, you all can't be out .” (PWID 12)

In terms of access to low barrier buprenorphine treatment, lack of face-to-face interactions reduced acceptability for some PWID and provider participants. For those PWID participants who still sought substance use disorder treatment in person, COVID-19 policies, particularly masking requirements, reduced acceptability. One PWID participant reported a history of kidnapping, and that wearing a mask was traumatizing for her, and masking requirements contributed to her decision to abandon a methadone program.

### Facilitators that increased acceptability of harm reduction services

In terms of other harm reduction services, trust in peers/community partners generally increased acceptability of peer support and SSP, so services that were able to continue operating were crucial. Some services actually increased outreach, or dedicated greater efforts to building relationships:

“I think we're really just trying to listen to the clients even more than we already have. […] we've always had a great staff, but I think they're really shining even more now. In the slower pace, you're really able to sit with folks more and hear them out and try to do things that you might not have otherwise had time for at one point […] Just let them know that while [this Site] doesn't look the same as it did three months ago, the spirit of [the Site] and our goal is still the same.” (CP 6)

Mobile SSPs also increased outreach. For some PWID, these new means of accessing safe injection/smoking supplies were more acceptable than previous options because they provided anonymity by operating in less populated, remote areas:

“It seems there's been this real shift that has been really effective. People have been really accessing services that way. I think partly just because there's more anonymity. You can show up. You're in, you're out.” (CP 3)

The mail delivery of equipment was also appealing to many participants due to perceived anonymity and thus increasing acceptability of services. Regarding MOUD treatment, some providers speculated that the inconsistency of drug supply/contaminated drug supply encouraged more people to seek treatment with MOUD, though we were unable to corroborate this in PWID interviews. Some clients did report that telemedicine visits for MOUD to be more acceptable than in-person visits, largely due to reduced travel time and costs. Finally, while social distancing and screening policies decreased the acceptability of sites for some clients, community partners reported that others appreciated the increased protection from COVID-19.

### Barriers that reduced harm reduction service accessibility

The ability of people to get to desired services was impacted in complicated ways, particularly as certain services transitioned from in-person provision to contact-less alternatives—counseling and treatment provided through telemedicine, and the mailing of injection supplies. Clients were differentially impacted depending on their access to smartphones, internet service, or a permanent address. Participants reported that people who did not have access to a phone and/or video calling, particularly those in rural areas, had difficulty accessing peer support groups. Similarly, lack of cell phone coverage and limited data plans were major barriers to low barrier telemedicine services in rural areas [37].

### Facilitators that increased accessibility to harm reduction services

Mobile units that distributed safe use equipment and naloxone increased SSP accessibility by bringing equipment closer to clients. A shift to telemedicine for MOUD treatment meant that patients with smartphones and access to Wi-Fi were able to more easily access treatment, without the need to physically travel to clinics [37]. The clinic that had set up outdoor services for patients also made space and equipment available for patients who were otherwise unable to access telemedicine appointments, which improved accessibility.

### Barriers that reduced availability of harm reduction services

Community partners at SSPs reported experiencing both a lack of personal protective equipment and shortages of safe injection supplies:

“We have had a hard time getting alcohol wipes, still waters, even fresh cotton, sterile cottons have been on back order…” (CP 1)

These issues limited what could be offered to clients:

“[Do they have everything that you do need?] Yes and no. They don't really have a lot of the pipes and stuff right now. […] And when it runs out, it runs out. You never know what they're going to have or how much; it's all about the donations and the volunteers. Sometimes it's clunky, sometimes there's nothing.” (PWID 3)

Some harm reduction suppliers also experienced staff shortages, especially earlier in the pandemic. Staff and volunteers were sometimes sick or did not want to risk exposure to the coronavirus. To minimize COVID-19 transmission, some on-site SSPs switched to pre-packaging equipment. However, this “one size fits all” change may have resulted in decreased availability of preferred supplies for some clients.

In the midst of this restricted supply, providers reported increased demand as some patients were using more drugs during the pandemic, possibly to cope with stress:

“I've certainly have had a number of my patients seem to be doing worse in terms of their substance use, that they're admitting that they're using more as a way to cope with stress.” (Pr 3)

Increased substance use meant increased demand for unused equipment in the setting of reduced access. Service providers often expressed concern about unsafe use:

“People [are] either using substances that they're not familiar with or purchasing substances from somebody who they're not familiar with and getting something that they don't know what it is or they thought it was something else. So we're seeing a lot of that. We're seeing a lot of people…using recklessly because they don't know when they're going to find something else.” (CP 3)

### Facilitators that increased availability of harm reduction services

During national shortages of medical supplies, outreach and community collaborations were important factors in increasing availability of basic services. Service providers reported increased communication between organizations to try and meet the needs of their clients:

“We have a supply distributor that's based out of Washington and certain things were just on back order and there was no getting around it. We're pretty much all getting together as the organizations. Like if one organization got 20 000 boxes of alcohol wipes, they were giving it out between the rest of us. […] Which gave all the organizations access, but limited access.” (CP 1)

Furthermore, service providers described leveraging connections among organizations to get access to naloxone and allow more distribution. SSP collaborations and sharing of equipment was thus an important facilitator that increased availability of safer use equipment for many SSP clients in rural areas. Relaxing rules and regulations around buprenorphine prescribing made low barrier buprenorphine treatment more available. These changes particularly allowed providers to prescribe buprenorphine via telemedicine at the first appointment, and thereby increased access [38].

### Reduced accommodation of harm reduction services to clients

Some SSPs were instructed to close early in the pandemic, which reduced access to services.

“The first time the cops closed us down, they closed us down saying that, we're not an essential service, you know what I mean? I mean it was a big hit just hearing that, because it would be an essential service if it was a bunch of privileged kids getting girl scout cookies at Walmart.” (CP 1)

While several mobile SSPs re-opened during the pandemic, these services still had limited hours. According to some participants, the lack of optimal service access led to more unsafe injection practices:

“… we only distribute once a week per town…I’ve had so many reports of people saying that they’re using supplies for money to get drugs because money’s harder to come by. And that leaves them without the safe using supply. And then, they end up sharing….” (CP 1).

Limited hours reduced access to SSPs and other basic services [39], particularly for people experiencing homelessness. COVID-19 screening was instituted to restrict entry to indoor sites delivering medical services. Lack of public restrooms also contributed to reduced access to basic services for unhoused participants or safe injection spaces.

### Increased accommodation of harm reduction services to clients

Community resilience, particularly the commitment of walk-in clinics to remain open despite risks and challenges, allowed service providers to better accommodate clients. The resilience of peer support organizations also increased adequacy of services; community partners reported lack of clear guidance early in the pandemic, however they adapted quickly to COVID-19 social distancing policies.

“We also kind of, on the fly, had to introduce a lot of COVID precautions that we really haven't been told to do…things like gloves, keeping people six feet apart from one another, asking folks not to touch the table and the stuff with it... And my coworker… even fashioned a $3 six-foot distance pole, a basket on the end of a six-foot pole.” (CP 2)

Despite the risk of potential COVID-19 exposure, several peer services remained open through phone or video calls. Some participants reported some peer support meetings still taking place in person, despite COVID-19 social distancing policies. SSP outreach workers continued to deliver services, despite some limitations such as less face-to-face interactions and physical distancing outdoors. No clients of mobile SSPs in rural areas reported any issues with accessing unused equipment.

“It would be easier at the end of the day for me to get COVID. But honestly, if we’re not helping people when they really need it the most, what are we even doing?”( CP 1)

### Barriers that reduced affordability of harm reduction services

Stricter adherence to the one-for-one needle exchange policy in the city of Portland reduced access to safer use equipment, as clients who appeared at the SSP without used needles to exchange were unable to acquire them. The enforcement of the one-for-one needle exchange policy also meant that some participants had to purchase equipment from pharmacies, which could also be cost prohibitive.

Several PWID participants reported higher drug costs (despite “low quality”/contaminated supply), as well as unemployment issues during the pandemic; if unable to afford travel costs to pharmacy or SSP, or if unable to afford equipment at pharmacies, financial challenges could affect their ability to access unused drug equipment and/or access low barrier treatment services.

### Facilitators that increased affordability of harm reduction services

A key facilitator that increased affordability, i.e., how clients perceived services, was the elimination of a statewide policy requiring one-for-one needle exchange at SSPs. Most SSPs adopted the change, particularly those operating in non-urban parts of Maine, meaning that people could freely access safe supplies without the need to carry used and therefore “illegal” equipment. While some PWID expressed concerns that needles would not be disposed properly in special containers installed throughout the city, most reported positive impacts of the change:

“The [SSP outreach workers] around here are pretty cool…they’ll give you as much as you need…unless they’re really, really short and they don’t have a lot of supplies that day, but usually they just say take as much as you want, as much as you need, give them to your friends, pass them out. They’re really…awesome.” (PWID 3)

Relaxation of the one-for-one rule and restrictions on sites for SSPs also allowed mobile SSPs more flexibility to supply equipment throughout the state. Being able to access free equipment from mobile SSPs, also mitigated travel costs and equipment costs at pharmacies or other sites. In addition, PWID participants accessing treatment using video calls also reduced travel costs and therefore increased affordability of treatment services.

### Cumulative impacts of the pandemic

Many participants reported that PWID lack safe housing or shelter options and experienced reduced access to a variety of public services. Changes in operating hours, available supplies and restrictive policies exacerbated the perceived loss of resources. Additionally, enforcement of public health policies throughout cities and towns that prevented people from congregating left many PWID expressing feelings of abandonment:

“I think the city, the police in particular, and city hall could do a lot to lift some morale of people that are sleeping in the park and that get kicked out. We woke up at eight this morning to police kicking on our tent, kicking it and saying, "Hey, wake up. You guys got to move. You're not allowed to be in all of Portland with your tent." I mean, what the hell are we supposed to do? Move to the next town? I mean, I just sat down for the whole day, and now we feel hopeless, and alone, and even further down.” (PWID 6)

## Discussion

In this study, we found that changes that occurred during the COVID-19 pandemic affected harm reduction services across many dimensions of access. Notably, we identified several facilitators and barriers to accessing harm reduction services during the COVID-19 pandemic that have policy and service delivery implications. Policies that resulted in the elimination of the one-for-one needle exchange, expansion of mobile SSP units, and permission of mail delivery of safer use equipment were particularly important in improving accessibility, availability, acceptability, and affordability of harm reduction services. On the other hand, modifiable policies, such as restrictions on drug, needle/syringe and other unused equipment possession, as well as the implementation of the one-for-one needle exchange policy, restricted harm reduction service availability, acceptability and affordability.

Harm reduction service changes that occurred during this study were similar to recent studies; SSPs, for example, adapted quickly, though still faced service disruption [14, 40]. Service changes to low barrier medication for opioid use disorder were similar to other settings; for example, the increased use of telemedicine. Telemedicine was not the focus of our study, however we did identify facilitators and barriers consistent with prior work that has pointed out both opportunities and disparities associated with telemedicine [41]. While telemedicine facilitated access to low barrier buprenorphine, as well as access to peer support services, for some participants, it was still a barrier for individuals lacking cell phone coverage in rural areas. Several service changes, such as masking requirements, physical distancing, and shelter and SSP protocols around COVID-19 screening and testing, were implemented to minimize COVID-19 transmission, however these changes also unfortunately reduced harm reduction service access for some participants.

Our results suggest that the one-for-one needle exchange policy, which reduced availability to and affordability of safer use equipment in this study, is a policy that should be permanently eliminated. Increased access to safer use equipment can improve injection practices and minimize adverse outcomes, such as injection drug-use associated infections [42, 43]. Notably, in urban areas where one-for-one exchange was more strictly enforced, PWID participants of on-site SSPs reported reduced availability of unused equipment. In contrast, PWID participants in rural areas, where the one-for-one exchange policy was not enforced, reported no issues with supply shortage. Affordability and acceptability also improved because of elimination of the one-for-one needle exchange policy- participants trusted SSP staff, and they did not have to spend extra money on safer use equipment at pharmacies, where they often reported feeling stigmatized. Particularly in rural areas, where secondary exchange (i.e., distribution of unused needles/syringes from one client to a network of people) commonly occurs, the elimination of the one-for-one needle exchange policy can facilitate access to safer use equipment [44]. Our study results also suggest that individuals in urban areas would also benefit from elimination of the one-for-one needle exchange policy, as several urban-based participants reported infections secondary to reusing equipment.

In our study, the combination of mail delivery of safer use equipment with expansion of mobile SSP units increased service access. These services are particularly important for rural areas where distance is a barrier to SSP use [17]. Mail delivery of equipment has shown promise in other areas of the USA, however funding and state-level policy changes may be necessary for expansion [45]. Similar to our findings, others have described how mobile SSP units reduce needle sharing and reuse of needles, facilitate referrals to treatment, while also providing essential services [46]. In our study, mobile SSP units increased availability, as well as acceptability for many participants. Accessibility to SSPs improved in rural areas because mobile SSP participants were able to avoid multiple visits to access on-site SSPs.

We found that allowing mobile units and mail delivery of equipment described above, as well as supporting policies that reduce stigma, such as drug decriminalization, may facilitate increased trust. Trust in community partners and providers and community resilience were also important facilitators to accessing harm reduction services during the pandemic. These findings are in line with prior work [47, 48]. Several community partner and provider participants reported that simply being present, open, and available enabled trust with PWID during the pandemic. On the other hand, we found that stigma and distrust were important barriers to accessing harm reduction services, which is consistent with other studies [49].

We also identified several barriers to accessing harm reduction services that are modifiable from a policy perspective. At the time of the study, due to state laws restricting the possession of more than one needle/syringe, as well as possession of drug of small amounts of drug, several participants reported harassment and other adverse outcomes from law enforcement. Lack of regulated, safe drug supply and the presence of new/unknown drug sources was raised as a serious concern due to overdose risk [50]. Given these barriers to safer use, drug decriminalization and legalization should be considered, as others have long been advocating [51].

There were several limitations to our study. Due to social distancing restrictions around COVID-19, interviews were conducted over the phone. In-person interviews allow more opportunities to build rapport with participants, facilitating additional information. Since this was not a longitudinal study, we did not build a relationship/rapport with PWID first, therefore it is possible some information was withheld due to distrust. Our recruiting methods favored PWID who were accessing harm reduction services or congregating in areas where our flyers were posted. Thus, our findings may not be generalizable to PWID disengaged from community services. Our sample was limited to people who speak English; therefore, we are missing perspectives from non-English speaking PWID. Since Maine is a state with predominantly (94.4%) white people [52], race/ethnicity information was not collected in order to protect confidentiality, especially in rural areas. Thus, we were not able to capture additional barriers related to race/ethnicity. With PWID interviews, recall bias with regards to service access during rapidly changing public health COVID-19 restrictions was another limitation. However, we tried to address this limitation through the inclusion of community partners and providers, who observe impacts across many clients and patients. Furthermore, we externally validated claims about policy changes through news reports and official websites and identified no inconsistencies. Finally, as our study was conducted in a rural state and an area with a relatively low incidence of COVID-19 cases compared to other regions, our results may not be generalizable to more urban settings and places with higher burden of COVID-19.

### Implications for practice

In this study, we identified facilitators and barriers to harm reduction service access in a rural state. Advocating for relaxed policies; specifically elimination of the one-for-one exchange policy, and allowance of mobile units and mail delivery of unused equipment, can facilitate increased access to harm reduction services. The use of telemedicine, particularly in rural areas, can also facilitate access to low barrier treatment for some PWID. Finally, strengthening support for SSPs should be a priority; SSPs were not only integral in making unused equipment accessible to PWID so they could use drugs safely, but they were also key in providing non-judgmental, innovative services to a population that is often otherwise neglected by society.

## Conclusions

Changes resulting from the COVID-19 pandemic have impacted many dimensions of access to harm reduction services among people who use drugs. Our study adds to the existing literature by identifying facilitators and barriers to accessing an array of harm reduction services in a rural context. We have included the perspectives of people who inject drugs, community partners and providers, all of whom have valuable insight on how to improve access to harm reduction services. Our results can inform policies and service delivery to maximally mitigate the negative impacts on people who use drugs, particularly in rural areas during, and beyond, the pandemic.

## Supplementary Information

**Additional file 1**. Interview guides.

**Additional file 2**. Additional study participant information.

## Data Availability

De-identified data can be made available upon request to corresponding author.
