# Supplementary material for: The impact of the COVID-19 pandemic on people who inject drugs accessing harm reduction services in an rural American state
Source: Harm Reduct J. 2022 Jul 22;19:80. doi: 10.1186/s12954-022-00660-2 (PMC9305035; doi:10.1186/s12954-022-00660-2)
Supplement: Supplementary file 1 — Additional file 1. Interview guides. [file 12954_2022_660_MOESM1_ESM.docx]

**Interview Guide for People Who Inject Drugs**

**Background:**

I’m going to start by asking you some questions about your life before the coronavirus epidemic.

1. How old are you?
2. Where in Maine do you spend most of your time?
3. What’s your current living situation? [Probe: living on own/with family/with roommates/halfway house].
   1. Has that changed at all during the last year? How so?
4. In the past year, how often have you used illegal or prescription drugs for nonmedical reasons?
   1. Are you currently using any substances? Which ones?
5. Are you currently involved in any form of treatment?

**Beliefs, Attitudes and Practices around COVID-19**

Now, I’d like to understand how things changed for you after the epidemic started, say after the stay at home order in mid-March. (Adapted from qualitative interview guide by Alex Bennett and Luther Elliott^[[1]](#footnote-1)^)

1. What have you heard about this coronavirus, or COVID-19?
2. How serious do you think this is? Tell me more.
   1. What kind of people do you feel are most at risk?
3. Do you feel like it’s a risk to you or people you know? [Probe: perceived risk]
4. How do you feel about the idea that everyone should avoid contact with other people?
5. Have you changed anything in your day to day life related to COVID-19?
   1. Where are you spending your time?
   2. Are you trying to stay 6 feet away from others?
   3. Anything else?
6. Can you talk to me a little about how you think the outbreak has affected your access to employment or informal wages? [Probe: In what ways is it harder or easier to make money or secure benefits?]
7. In what ways has the average day of substance use for you changed since the outbreak? [Probes: Stockpiling? Changed dosage? Other supplies? Emergency planning? Switching to other substances?]
8. Can you tell me about any changes in the environments where you use since the virus outbreak? [Probes: do you use in public places? Have the social settings for PWID/PWUO changed? Are there places where people still congregate and/or use together? Can you tell me about using public restrooms to inject/use?]
9. How has COVID-19 affected other people you know who use drugs?
10. How, if at all, has the virus changed your relationship with [other people who use drugs/in recovery]?
    1. With your family or other non-using friends?
    2. Can you tell me about any ways in which you feel you’re being treated differently?
    3. Or ways in which you’re treating other people differently (potentially because of fears of transmission)?
11. Can you talk a little about how the drugs you buy have changed, if at all, since the outbreak?
    1. Do you think the supply will be affected? Why or why not?
    2. Do you think prices will be affected? Why or why not?
    3. Do you think potency will be affected? Why or why not?
12. Can you talk about your access to safe use equipment, like syringes, cookers, or alcohol wipes?
    1. In what ways has access been impacted by the outbreak?
    2. What do you do when you can’t access this equipment? [Probe about sharing]
    3. What about access to equipment for using other drugs? [Probe about sharing]
13. Have you used a needle exchange program? [Clarify use before/after outbreak] Did anything about that program change after COVID-19?
    1. And did that have any effect on you? Could you still access it the same way? What was different? [Probes: One for one exchange, mobile units]
    2. What did you think of this change? [Probe for attitude]
    3. Thinking about after COVID-19, do you hope they keep this change or that the program goes back to the way it was before?
    4. [If less access] So what did you do? Were you able to find another way to get needles and gear [or other services, as applicable]?
    5. Is there anything you think the program should have done to help you meet your needs better with everything going on with COVID-19? What should they do? Why?
14. In what ways does coronavirus change how you think about overdose and overdose risk, if at all? [Probes: concerns about response times and willingness of peers/bystanders to intervene? Concerns about increases in solitary use?]
15. Can you tell me about how you think the coronavirus is affecting the way people think about and use naloxone?
    1. Can you tell me about your own experiences with naloxone since the outbreak? [Probe: would you feel confident using naloxone on someone overdosing? How about rescue breathing? Why or why not? What do you feel about the mouth guard provided in the standard blue-bag naloxone kit? How confident are you that it would protect you against the virus?]
    2. Can you tell me about any experiences of getting naloxone refills since the outbreak? [Probe: Stockpiling? Pharmacy availability via Medicaid? Lack of access via shuttered OOPPs? Fear of losing access if more agencies are shuttered?]
16. Can you talk to me about how the outbreak may have changed the way you feel about treatment?
    1. Are you more or less likely to seek buprenorphine or methadone treatment than before the outbreak? Why?
17. Are you confident you would be able to receive treatment if you wanted it? Why or why not?
18. Are you currently or have you previously been treated with MAT (buprenorphine, methadone, naltrexone)?
    1. Tell me about your experience with that.
19. Are currently using any treatment services, like a counselor or support groups? Which program(s) have you used?
    1. When did you use that program? For how long? [Probe to determine whether using it in time leading up to Covid-19]
    2. Did you think it was helping?
    3. [If no longer using] What happened?
20. [If applicable] You mentioned using X [specific treatment services noted above]. Did anything about that program change after COVID-19?
    1. Did it all change at once, or have there been more changes from beginning of the epidemic until now? Tell me about that.
    2. Did any of those changes have an effect on you? [Probe for impacts]
       1. Could you still access it the same way? What changed?
    3. Did they start doing anything through phone or video that used to be in person?
       1. Did you participate in any of that?
          1. [If not] Why not?
          2. [If yes] What did you think about that? Did you like it better or worse than meeting face-to-face?
          3. Would you want them to keep doing it this way after COVID-19 is over?
    4. Do you think any of these changes affected you in a bad way? Which ones? Why?
    5. Do you think any of these changes affected you in a good way? Which ones? Why?
    6. Thinking about after COVID-19, do you hope they keep this change or that the program goes back to the way it was before?
    7. Is there anything you think X [program being used] should have done to help you meet your needs better with everything going on with COVID-19? What should they do? Why?
21. Is there anything you think either the city or the state or even the federal government should have done in order to meet your needs during this time? What should be done?

**Impacts on Health**

1. Have you had any health issues (eg hospitalizations for IDU-related complications, overdose) since the epidemic started?
   1. [If applicable] How did this experience differ for you compared with ones you had prior to the epidemic?
   2. Have you heard about complications or problems that friends of yours have experienced as a result of the epidemic? Can you tell me about them?
2. Have you had any other health issues since COVID-19?
   1. What about your mental health?
3. Have you felt any more down or depressed since COVID-19 began?
   1. [If yes] How has this compared to before COVID-19?
   2. Can you think of any reason you might be feeling this way?
   3. What do you do to help deal when you’re feeling down or depressed?
4. Have you felt scared or anxious?
   1. [If yes] How has this compared to before COVID-19?
   2. Can you think of any reason you might be feeling this way?
   3. What do you do to help deal with these kind of feelings?
5. How has this whole coronavirus outbreak affected the way you feel about things in general?

Wrap-up

1. Those were most of my questions, but before we end, I’d like to make sure you’ve had an opportunity to discuss what you think is important. Thinking back to what we’ve talked about today, is there anything you’d like to clarify or emphasize?
2. And finally, we are trying to understand how social distancing is impacting the ability of PWID to access harm reduction and treatment. Can you think of anything else related to that goal which we haven’t talked about today?

**Interview Guide for Providers**

Background:

1. Where do you practice? [Probe for multiple locations, specific units]
   1. How long have you worked there?
2. In what ways (if any) have the changes due to concerns related to COVID-19 changed how you personally do your work (e.g. work at home, telemedicine)?
   1. Probe: Are there other policies, such as restrictions on certain medications, that have been introduced?
   2. Probe: Are you using telemedicine more than prior to March 18?
3. When working, how often do you see patients who inject illicit drugs? [repeat line of questioning for each clinical location, if multiple]
   1. While working, what is the most common reason you see people who inject illicit drugs?
      1. What other reasons do you see PWIDs?
   2. Have you noticed any changes in the number of PWIDs you see or the reasons they present since mid-March (or March 18), when social distancing measures were implemented?
      1. [If so] What do you think has led to that change?
   3. Have you noticed any changes in the kinds of health issues PWIDs have been presenting with recently, as compared to before March 18, when social distancing measures were implemented?
      1. Have you noticed any changes in the number of complications or overdoses for PWID since the epidemic started?

Effects of efforts to contain Covid-19 on patients and those who inject drugs:

1. What are some specific ways that efforts at your clinic/hospital/etc. [use appropriate term] to contain the spread of the novel coronavirus could be affecting patients (in general)?
   1. Can you think of ways it may be affecting patients who inject drugs specifically?
   2. Can you think of any examples where a PWID was negatively impacted by such efforts?
   3. [If participant stated they were using telemedicine and did not already mention impacts] Have you used telemedicine more now than you have in the past?
      1. Can you tell me about your experiences using telemedicine?
      2. How does treating PWID over telemedicine compared to face-to-face visits? What’s easier? What’s harder?
      3. Do you think you’d like to use telemedicine more or less in the future? Why or why not?
      4. How might telemedicine be used to expand access to care for PWIDs outside of the COVID pandemic?
2. Are you involved specifically with harm reduction for PWIDs or substance use treatment?

[If yes, ask the following:]

- - 1. How are you involved specifically? For example, do you provide counseling, prescribe for treatment…?
    2. How (if at all) has that work (on HR or SUDT) changed due to concerns about Covid-19?
       1. Why specifically was that modification made (Required by law? Local decision?)?
       2. [Probe if not mentioned] Any additional use of telemedicine?
    3. Have you noticed any changes in how PWIDs have used those services since social distancing restrictions went into effect? (e.g. more/less utilization, different people, etc). What are those changes?
       1. What do you think led to those changes?
       2. Based on what you’ve seen, have PWID’s been able to access that activity/service in the same way since Covid-19? [If not] What’s different?
       3. [If modifications] How (if at all) do you think those changes are impacting individuals who typically use X?
          1. Probe: Impacts on drug use, infection (or potential), ODs, mental health, other?
          2. Probe: Are there positive impacts?
    4. Have you or others been able to mitigate these impacts? How?
    5. Do you think any of these changes will be maintained after the pandemic? (Which ones? Why?)

Broader and Other Impacts

1. Now if I could ask you to think more broadly, drawing on your experience and knowledge as a provider, how do you think efforts to slow the spread of Covid-19 are affecting the health of people who inject drugs in your area?
   - 1. Probe: Impacts on drug use patterns, infections, ODs, mental health, other?
     2. Probe: Any positive impacts?
   1. What about people who are homeless? How do you think these changes for COVID-19 are affecting them?
      - 1. Are people still able to access these services in the same way? What has changed?

Wrap-up

1. Those were most of my questions, but before we end, I’d like to make sure you’ve had an opportunity to discuss what you think is important. Thinking back to what we’ve talked about today, is there anything you’d like to clarify or emphasize?
2. And finally, we are trying to understand how social distancing is impacting the ability of PWID to access harm reduction and treatment. Can you think of anything else related to that goal which we haven’t talked about today?

**Interview Guide for Community Partners**

Background:

1. For how long (elicit specific unit of time) have you been working with people who inject drugs altogether? In Maine specifically?
2. Where in Maine do you predominantly work? (Do you mainly interact with PWID living in urban/rural areas?)
3. With what program(s) are you affiliated? What is your role in that/those program(s)?
4. In what ways (if any) hasCOVID-19 changed how you personally do your work (e.g. work at home, frequent handwashing, using PPE, healthy hand-offs, etc)?

Specific Activities of Involvement:

1. What specific activities are you involved with that either address harm reduction for PWID or substance use disorder treatment? (Note each)

[If multiple, then for each activity noted above, ask the following:]

- - 1. And what is the goal of activity X? (probe for potential health impacts)
    2. Have you experienced any shortages in staff related to COVID-19?
    3. Have you noticed any changes in how that activity/service has been used since the coronavirus outbreak started? What changes have you noticed? ? (e.g. more/less utilization, different people, etc). What are those changes?
       1. What do you think led to those changes?
       2. How has utilization changed over the course of the epidemic?
       3. Have PWID’s been able to access that activity/service in the same way since Covid-19? [If not] What’s different?
          1. Have you seen access change over the course of the epidemic? How?
    4. Was X officially modified in any way due to Covid-19?
       1. Why specifically was that modification made? Required by law? Local decision? [If not answered] What was the justification for the modification?
       2. [If modifications] How (if at all) do you think those changes are impacting individuals who typically use X?
          1. Probe: Impacts on drug use, infection (or potential), ODs, mental health, other?
          2. Do you think any of these changes could have a positive effect on PWIDs?
    5. What have you or others (or your agency) done to try to mitigate the impact of the changes on PWID?
       1. How effective have these modifications been?
       2. How have your mitigation strategies changed over time/ through the epidemic?
       3. Knowing what you know now, would you have done anything differently? Why or why not?
       4. Do you think any of these modifications or adaptations could be maintained after the pandemic? (Which ones? Why?)

Broader and Other Impacts

1. Broadly speaking, how are social distancing efforts affecting people who inject drugs in your area?
   - 1. Are they impacting (other) harm reduction services for PWID? [If so, which ones? How?]
        1. Are people still able to access these services in the same way? What has changed?
     2. Are they impacting (other) treatment services for PWID? [If so, which ones? How?]
        1. Are people still able to access these services in the same way? What has changed?
     3. What about people who are homeless? How do you think these changes for COVID-19 are affecting them?
        1. Are people still able to access these services in the same way? What has changed?
2. Do you think that social distancing or other changes due to Covid-19 are impacting the health of PWID in your area? [If so, in what way? What have you seen?]
   - 1. Probe: Impacts on drug use patterns, infections, ODs, mental health, other?
     2. Do you think any of these changes might have positive effects (aside from reducing spread)?
3. Thinking about the changes made to these programs due to COVID-19, do you think any will be maintained after the pandemic? (Which ones? Why?)

Wrap-up

1. Those were most of my questions, but before we end, I’d like to make sure you’ve had an opportunity to discuss what you think is important. Thinking back to what we’ve talked about today, is there anything you’d like to clarify or emphasize?
2. And finally, we are trying to understand how social distancing is impacting the ability of PWID to access harm reduction and treatment. Can you think of anything else related to that goal which we haven’t talked about today?

1. <https://projectreporter.nih.gov/project_info_description.cfm?aid=9579673&icde=40831481> Their interview guide can be found here: https://clelandcm.github.io/COVID19-Interview-Items/COVID-Items.html?fbclid=IwAR2W2wZTQPt_qBSdWY_w3ek-DPY0c2S4ytUh8Z9c4n2BwZ3iXkFe_ovXLec [↑](#footnote-ref-1)
