# Supplementary material for: The impact of the COVID-19 pandemic on people who inject drugs accessing harm reduction services in an rural American state
Source: Harm Reduct J. 2022 Jul 22;19:80. doi: 10.1186/s12954-022-00660-2 (PMC9305035; doi:10.1186/s12954-022-00660-2)
Supplement: Supplementary file 2 — Additional file 2. Additional study participant information. [file 12954_2022_660_MOESM2_ESM.docx]

**Additional file 2: Table S1. Additional Study Participant information**

| **Participants** | **Additional details** |
| --- | --- |
| People who inject drugs (n=18) | - 89% (n=16) reported accessing SSPs* for supplies - 61% (n=11) unhoused at time of study interview |
| Community partners (n=9) | - Included participants from 2 sanctioned, on-site SSPs serving urban regions - Included participants from 1 SSP (with multiple sites) delivering supplies in rural areas - Included participants from 1 unsanctioned SSP with mobile distribution in rural areas - All with significant experience working with people experiencing homelessness (i.e emergency shelter staff, outreach to unhoused people living in tents/on streets) - 33% (n=3) worked as peers and/or with peer support organizations |
| Providers (n=9) | - 22% (n=2) worked at outpatient clinic that provided low barrier buprenorphine treatment - 11% (n=1) worked at emergency department that provided low barrier buprenorphine treatment |
